# Genetic characterization of human adenoviruses in patients using metagenomic next-generation sequencing in Hubei, China, from 2018 to 2019

**DOI:** 10.3389/fmicb.2023.1153728

**Published:** 2023-03-16

**Authors:** Bin Fang, Juan Lai, Yongfeng Liu, Tian-tian Yu, Xiao Yu, Xiang Li, Lijun Dong, Xin Zhang, Wei Yang, Qin Yan, Lei Sun, Lin-lin Liu

**Affiliations:** ^1^Hubei Provincial Center for Disease Control and Prevention, Institute of Health Inspection and Testing, Wuhan, China; ^2^GeneMind Biosciences Company Limited, Shenzhen, China; ^3^School of Public Health, Department of Nutritional Hygiene and Toxicology, Wuhan University of Science and Technology, Wuhan, China

**Keywords:** human adenovirus, metagenomic next-generation sequencing, phylogenetic analysis, recombination analysis, genetic characterization

## Abstract

**Objectives:**

This study aimed to characterize the genomic epidemiology of human adenoviruses (HAdVs) in Hubei, China, using metagenomic next-generation sequencing (mNGS).

**Methods:**

In total, 25 HAdV-positive samples collected from 21 pediatric patients were sequenced and subjected to mNGS using the NextSeq 550 and GenoLab M sequencing platforms. The metagenomic data were assembled *de novo* for molecular typing, phylogenetic and recombination analyzes.

**Results:**

We assembled 50 HAdV genomes, 88% (22/25) genomes from GenoLab M, and 84% (21/25) genomes from NextSeq 550 have perfect alignments to reference genomes with greater than 90%. The most fully assembled 25 genomes were categorized into 7 HAdV genotypes, the most abundant of which were HAdV-B3 (9/25) and HAdV-C2 (6/25). Phylogenetic analyzes revealed that the newly isolated HAdV-B3 strains diverged into separate clusters according to their genotypes. Vigilance is needed that HAdV-B3 isolates have begun to form new distinct clusters. High nucleotide identity was observed in the whole genome level within the same HAdV genotypes, while marked differences of three capsid genes across HAdV genotypes were noted. The high nucleotide diversity regions were concordant with the reported hypervariable regions. Further, three recombinant strains were identified: S64 and S71 originated from the parental strains HAdV-B14 and HAdV-B11, and S28 originated from HAdV-C1, HAdV-C5, and HAdV-CBJ113. GenoLab M and NextSeq 550 showed comparable performance with respect to data yield, duplication rate, human ratio, and assembly completeness.

**Conclusion:**

The sequencing quality and assembly accuracy showed that mNGS assembled genomes can be used for subsequently HAdV genotyping and genomic characterization. The high nucleotide diversity of capsid genes and high frequency of recombination events has highlighted the necessity for HAdV epidemiological surveillance in China.

## 1. Introduction

Human adenoviruses (HAdVs) are nonenveloped, double-stranded, linear DNA viruses belonging to the genus *Mastadenovirus*, family *Adenoviridae* (Davison et al., 2003). The adenovirus genome is approximately 35 kb long and enclosed in an icosahedral capsid along with core proteins. The icosahedral capsid mainly comprises the capsid proteins hexon, penton, and fiber, which are the principal mediators of the virus–host cell interactions, conventionally used for serum neutralization and genotype identification (Davison et al., 2003; Walsh et al., 2009). Currently, 111 HAdV genotypes have been assigned according to the classification criteria of the Human Adenovirus Working Group (http://hadvwg.gmu.edu/, March 2022 update). These genotypes are classified into seven species (A–G) based on their biochemical properties, DNA homology, and genomic sequences. HAdVs are highly contagious pathogens that cause various diseases owing to variations in tissue tropism and virulence. Species B, C, and E mainly infect the respiratory tract; species D targets the conjunctiva; and species A, F, and G prefer the gastrointestinal tract (Jones et al., 2007; Robinson et al., 2008; Chen et al., 2016).

Epidemiological studies have confirmed that HAdV infections occur in all age groups with generally mild clinical consequences, while children, military recruits, immunocompromised patients, and people with underlying diseases are at a higher risk for developing severe diseases (Kolavic-Gray et al., 2002; Lion, 2014; Zou et al., 2021). Among the HAdV genotypes, HAdV-1 to 7 (HAdV-B3, B7, C1, C2, C5, C6, and E4) are the most frequently detected, accounting for >80% of all HAdV respiratory tract infections in pediatric patients (Garnett et al., 2002; Sirena et al., 2005; Huang et al., 2021). The predominant epidemic strains HAdV-B3 and B7 exhibit high virulence and are associated with severe clinical manifestations, such as residual lung damage and even fatal outcomes in infants and children (Sirena et al., 2005). Comparatively, HAdV-C infection may be mild, self-limiting, and most commonly detected in infants aged <2 years (Yang et al., 2019). Interestingly, HAdV-E4 is the only type classified within species E and associated with respiratory tract and ocular infections. Based on previous reports, the HAdV-E4 outbreak typically and inexplicably occurred in United.States military settings (Potter et al., 2012) with a low frequency in pathogen surveys of the civilian population (Zhang et al., 2019).

Conventional serology-based typing (e.g., serum neutralization assays and hemagglutination-inhibition tests) is used for the diagnosis and epidemiological studies of HAdVs. However, serologic typing methods are laborious and difficult, particularly when they lack type-specific antisera. The development of genomics and bioinformatics has facilitated adenovirus classification and revolutionized the conventional serology-based typing method (Seto et al., 2011; Ismail et al., 2018). Whole-genome data enables a thorough and high-resolution characterization of HAdVs according to their types, potential morbidity and mortality profiles, and molecular evolution. Sequence-based phylogenetic analysis is a valuable tool for the epidemiological investigation of HAdVs outbreaks. It is widely used for typing the HAdVs genotypes that are currently circulating (Madisch et al., 2005), detecting coinfection with multiple HAdV species (Vora et al., 2006), and, most importantly, discovering new recombinant strains (Lukashev et al., 2008; Walsh et al., 2009). Homologous recombination is one of the crucial factors driving the molecular evolution of HAdVs and has been previously identified in various HAdVs species (Ismail et al., 2018; Yang et al., 2019). Through intra-and interspecies recombination, HAdVs may evolve faster and generate novel genotypes with increased fitness and broader cell tropism (Robinson et al., 2013). With respect to species B, HAdV-B55 (FJ643676) is a well-studied recombinant strain that evolved from intertypic recombination between HAdV-B14 (AY803294.1) and HAdV-B11 (AF532578.1) (Yang et al., 2009; Walsh et al., 2010). HAdV-B79 (LC177352.1), a novel genotype, is a recombinant strain originating from HAdV-B34 (AY737797), HAdV-B11 (AY163756), and HAdV-B14 (JQ824845) (Yoshitomi et al., 2017). For species C, three recombinant strains, BJ04 (MF315028), BJ09 (MF315029), and CBJ113 (KR699642), have been confirmed through genome-based phylogenetic and recombination analyzes. CBJ113 was characterized by recombination among HAdV-C2 (AC_000007.1), HAdV-C6 (HQ413315.1), HAdV-C1 (AC_000017.1), HAdV-C5 (AC_000008.1), and HAdV-C57 (HQ003817.1) sequences. BJ04 was found to share sequences with the parental strains HAdV-C1 (JX173086), HAdV-C2 (NC_001405), and HAdV-6 (LC068718), whereas BJ09 shared sequences with HAdV-C1 (JX173083), HAdV-C5 (KF268199), and CBJ113 (KR699642) (Wang et al., 2016; Mao et al., 2017). Except for intraspecies recombination, HAdV can evolve through interspecies recombination, as evidenced by HAdV-E4 (AY599837), owing to the recombination between HAdV-B16 (AY601636) and the simian adenovirus (SAdV) SAdV-E26 (FJ025923) (Dehghan et al., 2013). Statistically, nearly all newly discovered and identified genotypes are recombinants since HAdV-52 (DQ923122) (Wu et al., 2022).

Although viral whole-genome sequencing (WGS) is a valuable tool for HAdV research, it is not feasible for routine large-scale molecular epidemiological monitoring, as virus isolation and culture are time-consuming and limited by reagent availability (Zhu et al., 2009; Xie et al., 2013). Moreover, WGS is strenuous for rapidly identifying pathogens during outbreaks due to the long sequencing time. Conversely, metagenomic next-generation sequencing (mNGS) is a promising tool for rapid, culture-free pathogen surveillance and molecular epidemiology. It also enables coinfection detection without prior knowledge of the etiological agent (Miller et al., 2013; Liu et al., 2022). mNGS has proven to be successful in pathogen identification, coinfection detection, and epidemiological studies of the HAdV-55 outbreak in 2019 (Li P. et al., 2021), as well as the HAdV-7 outbreak during 2018–2019 in Hubei Province, China (Li Y. et al., 2021). Recently, mNGS significantly contributed to the rapid identification of severe acute respiratory syndrome coronavirus 2 (SARS-CoV-2) during the coronavirus disease 2019 outbreak in Wuhan, China, demonstrating its utility in the early stages of novel pathogen discovery (Chen L. et al., 2020).

In this study, we evaluated the utility of mNGS for characterizing HAdV infection in Hubei, China. Twenty-five HAdVs samples were sequenced using the NextSeq 550 and GenoLab M sequencing platforms. We determined the evolutionary relationships of the strains and potential recombination events through molecular typing and genome characterization.

## 2. Materials and methods

### 2.1. Ethics statement

This was a retrospective observational study based on the influenza surveillance system of Hubei Province. All the data in the surveillance system were anonymized. Approval for this study was obtained from the research ethics board of the Hubei Provincial Center for Disease Control and Prevention, and the requirement of obtaining informed consent was waived.

### 2.2. Sample collection and HAdV infection confirmation

Four hundred influenza-like nasopharyngeal swab specimens were collected using the YOCON Virus Sampling Kit MT0301-1 (YOCON Biology, Beijing, China) from patients in the influenza surveillance system of Xianning, Hubei Province, from 2018 to 2019. The KingFisher Flex platform (Prefill Viral Total NA Kit 2×96 preps, KFRPF-805296) was used for large-scale DNA extraction from the 400 swab specimens. After screening for common viral respiratory pathogens, 21 HAdV-positive patients were enrolled. HAdV infection was confirmed *via* polymerase chain reaction (PCR) using the AgPath-ID™ One-Step RT-PCR Reagent (Thermo Fisher Scientific, United States) by amplifying the hexon gene using the forward primer 5′-GCCACGGTGGGGTTTCTAAACTT-3′, the reverse primer 5′-GCCCCAGTGGTCTTACATGCACATC-3′, and the TaqMan probe 5′-TGCACCAGACCCGGGCTCAGGTACTCCGA-3′. The PCR conditions were as follows: denaturation at 95°C for 10 min, followed by 45 cycles at 95°C for 10 s, 55°C for 10 s, 65°C for 60 s, and signal detection at 65°C (Yang et al., 2021). Virus load was determined using cycle threshold (CT) values as a semi-quantitative indicator of viral titer (Brown et al., 2016). Four samples were cultivated as positive controls in HEp-2, a human laryngeal carcinoma cell line, for virus isolation and propagation.

### 2.3. DNA extraction from clinical nasopharyngeal swabs

DNA was directly extracted from the nasopharyngeal swabs using the EZ1 Virus Mini Kit (Qiagen, Hilden, Germany) according to the manufacturer’s instructions. DNA concentrations were quantified using the Qubit dsDNA HS Assay Kit and Qubit 4.0 fluorometers (Thermo Fisher Scientific, United States). The extracted DNA was stored at −80°C for subsequent mNGS.

### 2.4. Viral cultivation and viral DNA extraction

HEp-2 cells were inoculated with four nasopharyngeal swabs and cultured in Dulbecco’s Modified Eagle Medium supplemented with 100 IU/ml penicillin, 100 μg/ml streptomycin, and 2% (v/v) fetal bovine serum. The cell culture supernatant was collected and cell lysis buffer was added to it (three freeze–thaw cycles) for viral genomic DNA extraction when a 90% cytopathic effect (CPE) was observed. Similarly, the EZ1 Virus Mini Kit (Qiagen, Hilden, Germany) was used for viral DNA extraction. The resulting DNA concentration was measured using the Qubit™ dsDNA BR Assay Kit equipped with a Qubit™ 4 Fluorometer (Thermo Fisher Scientific, United States). DNA was stored at −80°C for library construction.

### 2.5. Library preparation and sequencing

DNA libraries were prepared using the VAHTSTM Universal DNA Library Prep Kit for Illumina® V3 (Vazyme, China) following the manufacturer’s instructions. The DNA samples were fragmented and ligated to adapters after being end-repaired and A-tailed. Next, the fragmented DNA was amplified and purified using the 0.8x Agencourt® AMPure XP bead purification kit (Beckman Coulter, United States). The insert sizes were determined using the Agilent 2,100 Bioanalyzer (Agilent Technologies, United States). Finally, the libraries were quantified using the Qubit™ dsDNA HS Assay Kit (Thermo Fisher Scientific, United States). One library for each sample was split into two copies, and each copy was sequenced using the Illumina NextSeq 550 or GeneMind GenoLab M platform, with a 150-cycle, single-end, high-output sequencing mode. Depending on the viral load (CT) in different samples, different read numbers (CT < 25, 10 M; 25 ≤ CT < 30, 30 M; and CT ≥ 30, 50 M) are recommended (Huang Z.-D. et al., 2020).

### 2.6. Raw data processing

SOAPnuke v2.1.6 was used to filter raw data from the abovementioned two platforms as follows: (1) reads with ≥60 bp with a quality score of <20, (2) reads with ≥1 N base, and (3) reads with an overlap of ≥8 bp with adapters. The trimmed reads were aligned to the human reference genome (GCF_000001405.40_GRCh38.p14) using Bowtie2 (version 2.4.5) and filtered to obtain clean data.

### 2.7. Species annotation

Kraken 2 (version 2.1.2; confidence: 0.7) was used to assign taxonomic labels to shotgun metagenomic DNA sequences using the Kraken standard reference database^1^, which includes archaeal, bacterial, viral, plasmid, human, and UniVec_Core sequences. To determine the applicability of single-end 75-bp sequencing mode in mNGS research, we subsampled the data by randomly extracting the 75 bp reads using seqtk (1.3-r117-dirty). Subsequently, species annotation was performed using Kraken 2.

### 2.8. Genome assembly, genotyping, and annotation

The 50 clean data sets (25 for NextSeq 550 and 25 for GenoLab M) were individually assembled *de novo* using the MEGAHIT v1.2.9 software. The Redundans pipeline was used to integrate the resulting contigs and scaffolds. Next, the assembled sequences were aligned to a curated reference list of complete adenovirus genome sequences using Minimap2 v2.24-r1122 to produce consensus sequences for each sample from the two platforms. We mapped the consensus sequences to the closest reference sequence to identify the HAdV types. The reference dataset consisted of 301 sequences obtained from two sources: GenBank accessions nos. MW686757–MW686857 and “Adenovirus AND srcdb_refseq” in the NCBI database. Following a visual inspection and comparison of the total length of the consensus sequences between the two platforms, we selected the longer one to perform gene prediction. The annotation was performed based on HAdV reference genome annotations using VIGOR3.

### 2.9. Phylogenetic analysis

Multiple sequence alignments for phylogenetic analysis, including nucleotide and protein sequences, were performed using the ClustalW program of MEGA11.0 (Molecular Evolutionary Genetics Analysis version 11). Considering that the unmatched fragments could contribute significantly to an error in detecting actual evolutionary divergence or sequence similarity, discretion was required by trimming the 5′ and 3′ ends of the mapped reads to generate a blunt end after alignment. The test “Find Best DNA/Protein Models (ML)” was performed to select the most appropriate evolutionary model for our phylogram. Based on the results of the previous step, phylogenetic trees were constructed using the maximum likelihood method with the bootstrap test of phylogeny with 1,000 replicates. Genetic distances were computed in MEGA11.0 and shown in the phylogenetic trees.

### 2.10. Recombination analysis

Potential genomic recombination events were identified based on the sequence alignment results from MEGA11.0. SimPlot (version 3.5.1) was further used to verify the potential recombination events. Bootscan analysis and SimPlot were performed with default parameters. Breakpoint sites were estimated according to the results of Bootscan and SimPlot analyzes.

### 2.11. Nucleotide identity and diversity analysis

The nucleotide identity of the newly assembled draft genomes and the HAdV reference genomes was calculated using FastANI (version 1.33, https://github.com/ParBLiSS/FastANI) by alignment-free computation of the average nucleotide identity (ANI). FastANI was performed with a fragment length of 500 bp for ANI pairwise values calculation. Then, the output triangular matrix data was used to draw a violin plot. Nucleotide diversity (π) plots of *penton*, *hexon*, and *fiber* genes were constructed using the DNA Sequence Polymorphism software (DnaSP v6.12.03, http://www.ub.edu/dnasp/) with a 100 nucleotide sliding window and 25 nucleotide step size.

## 3. Results

### 3.1. Patient characteristics

Twenty-one patients exhibited acute respiratory tract infection symptoms were enrolled in this study. Some information for three patients was missing. Among the other 18 HAdV-positive patients, 13 (72.22%) were male and 5 (27.78%) were female, resulting in a male: female ratio of 2.6:1. The age of the patients ranged from 1 to 11 years (median age, 3.86 years). The mean Ct value for the 25 samples was 21.19 (range, 15.56–32.13; Supplementary Table S1).

### 3.2. Data quality and assembly results

After data trimming and removing the human reads, we compared the sequencing quality of the NextSeq 550 and GenoLab M platforms. Although there were fluctuations between samples, we observed comparable performance between the two platforms in terms of data yield, GC content, duplication rate, and human ratio (Supplementary Table S2). Notably, GenoLab M showed remarkable advantages with respect to Q20 and Q30 percentage compared with NextSeq 550 (97.16% vs. 89.91 and 92.8% vs. 83.86%, respectively).

Subsequently, we assessed the *de novo* assembly results of HAdVs originating from GenoLab M by comparing them with those originating from NextSeq 550. Table 1 and supplementary Table S3 shows the detailed assembly statistics for the 25 samples, including the standard assembly metrics evaluating the continuity of whole-genome assembly. In most samples, contig numbers, N50, N90, maximum contig, and genome coverage were comparable between GenoLab M and NextSeq 550; however, for a few samples, such as S16-C1, S28, and S50, we found minor differences. The assembly accuracy was evaluated through the aligned percentage of the assembly to the reference viral genomes. For GenoLab M, 22/25 samples showed high alignments with greater than 90%, for NextSeq 550, the ratio was 21/25. Overall, the high accuracy and integrity of the assembly indicated the usability of mNGS for subsequent genomic characterization.

**Table 1:** Assembly statistics of 25 samples sequenced via two platforms.

	Reference	Reference length (bp)	Aligned base/reference (%)		Contig number		Largest contig length (bp)		Total length (bp)		N50 (bp)		N90 (bp)	
Sample			GL	NS	GL	NS	GL	NS	GL	NS	GL	NS	GL	NS
S11	MW816005.1	35,226	99.53	99.59	1	1	35,215	35,209	35,215	35,209	35,215	35,209	35,215	35,209
S15	MT424875.1	35,994	88.75	88.64	1	1	36,070	36,044	36,070	36,044	36,070	36,044	36,070	36,044
S16-C1	MW748645.1	35,255	99.62	99.56	1	2	35,423	19,799	35,423	35,262	35,423	19,799	35,423	15,463
S16	MW748645.1	35,255	99.66	99.66	1	1	35,277	35,277	35,277	35,277	35,277	35,277	35,277	35,277
S21	MW748657.1	35,255	99.49	99.60	1	1	35,292	35,397	35,292	35,397	35,292	35,397	35,292	35,397
S28-C1	MF315029.1	35,958	98.65	98.60	1	1	35,952	36,019	35,952	36,019	35,952	36,019	35,952	36,019
S28	MF315029.1	35,958	98.51	98.54	4	1	11,907	35,985	36,158	35,985	11,581	35,985	9,394	35,985
S3	KF006344.1	35,960	97.04	98.63	5	2	14,957	25,385	35,357	35,871	9,912	25,385	4,246	10,486
S33	MW748657.1	35,255	99.50	99.60	1	1	35,284	35,292	35,284	35,292	35,284	35,292	35,284	35,292
S4	KF006344.1	35,960	98.79	98.79	1	1	35,780	35,780	35,780	35,780	35,780	35,780	35,780	35,780
S41	MW748657.1	35,255	99.53	99.77	1	1	35,260	35,363	35,260	35,363	35,260	35,363	35,260	35,363
S43-C1	KF006344.1	35,960	79.12	98.49	11	3	9,835	12,959	29,236	35,983	2,936	12,407	1,455	10,617
S43	KF006344.1	35,960	98.67	98.68	1	1	35,796	35,796	35,796	35,796	35,796	35,796	35,796	35,796
S48	MK041241.1	35,951	94.51	94.64	2	1	29,692	35,907	35,929	35,907	29,692	35,907	6,237	35,907
S5	MW748654.1	35,264	99.38	99.44	3	2	15,069	24,507	35,319	35,318	10,860	24,507	9,390	10,811
S50-C1	MW748657.1	35,255	99.44	99.33	2	3	24,287	19,818	35,264	35,335	24,287	19,818	10,977	3,605
S50	MW748657.1	35,255	99.51	99.60	2	1	19,728	35,291	35,246	35,291	19,728	35,291	15,518	35,291
S55	MT263140.1	35,935	97.01	97.09	7	6	10,569	11,276	36,660	36,672	8,296	10,953	4,321	2,890
S58	MF315029.1	35,958	92.12	81.51	10	10	6,037	8,141	35,177	36,316	4,468	4,756	1,881	2,294
S59	MW748672.1	35,256	99.52	58.88	1	1	35,371	35,368	35,371	35,368	35,371	35,368	35,371	35,368
S60	MN513344.1	35,986	78.70	81.78	13	10	4,155	7,430	31,084	31,526	2,852	4,620	1,247	1,727
S63	MF681662.1	35,805	92.37	92.54	3	3	18,579	32,250	36,227	35,874	18,579	32,250	15,191	2,073
S64	KX289874.1	34,755	99.45	99.26	1	2	34,724	33,712	34,724	34,737	34,724	33,712	34,724	33,712
S71	KX289874.1	34,755	99.43	99.32	1	1	34,716	34,821	34,716	34,821	34,716	34,821	34,716	34,821
S9	MZ151863.1	35,936	98.04	97.93	1	1	36,035	35,990	36,035	35,990	36,035	35,990	36,035	35,990

GL, GenoLab M.

NS, NextSeq 550.

N50 size was calculated by arranging all sequences and then adding the lengths of sequences from the longest to the shortest until the summed length exceeded 50% of the total length of all sequences.

N90 is similarly defined as N50.

### 3.3. Interplatform consistency in microbial species annotation

**Figure 1:**
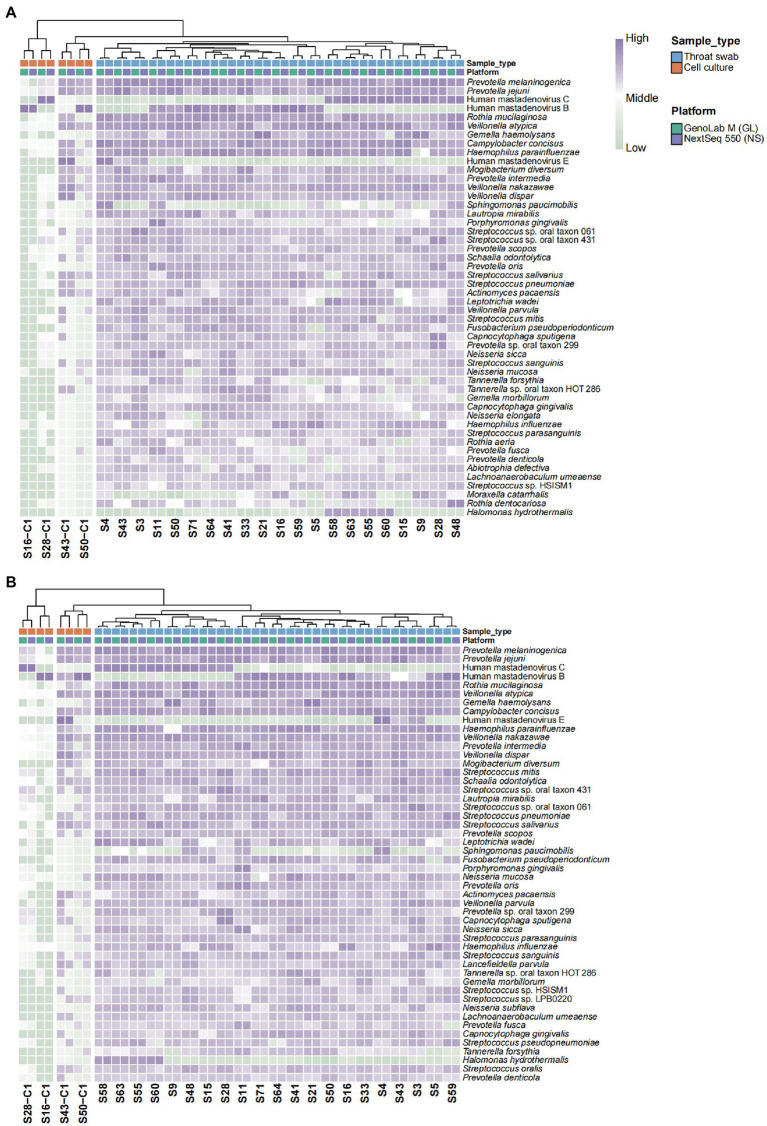
Heat map showing abundance clustering of the top 50 species. **(A)** SE150 and **(B)** SE75 sequencing mode. *Z* values represent the corresponding value of the heat map, which were obtained after normalization to the relative abundance of the species in each row. The color gradient from green to purple indicates low to high relative abundance. The x-axis displays the samples and groups and the y-axis represents the species annotation information. Horizontal clustering indicates the similarity of species richness in different samples.

To further explore the interplatform consistency in the detection accuracy of pathogens, species annotation and relative abundance were compared. The heat map showed remarkable consistency in species types and abundance across the two platforms (Figure 1A). The cell culture and nasopharyngeal swab samples were clustered into three branches. HAdVs of different types mainly dominated the cell culture samples. The S43-C1 sample also exhibited a high relative abundance of the bacterial genus *Veillonella* and *Prevotella*. In the nasopharyngeal swab samples, the distribution of dominant species detected in the same sample *via* two platforms showed high consistency and they were characterized by their respective subclusters. Compared with the cell culture samples, the clinical samples unsurprisingly revealed a wider variety of pathogens in addition to HAdVs, and the abundance of the genus *Prevotella*, *Veillonella*, and *Streptococcus*. The correlation matrices for the GenoLab M and NextSeq 550 platforms with respect to species abundance exhibited a high correlation coefficient (approximately 1.0; Supplementary Figure S1A). Consistent results were obtained for the subsampled data in 75-cycle (SE75) sequencing mode for species annotation and correlation (Figure 1B and Supplementary Figure S1B). Overall, the performance of GenoLab M was consistent with that of NextSeq 550 in mNGS for pathogen detection. The randomly intercepted SE75 sequencing data showed excellent reproduction of the results for the SE150 data, indicating the applicability of short-read-length sequencing in mNGS, which can effectively reduce sample turnaround time.

### 3.4. HAdV genotyping and reference-independent whole-genome phylogenetic analysis

**Figure 2:**
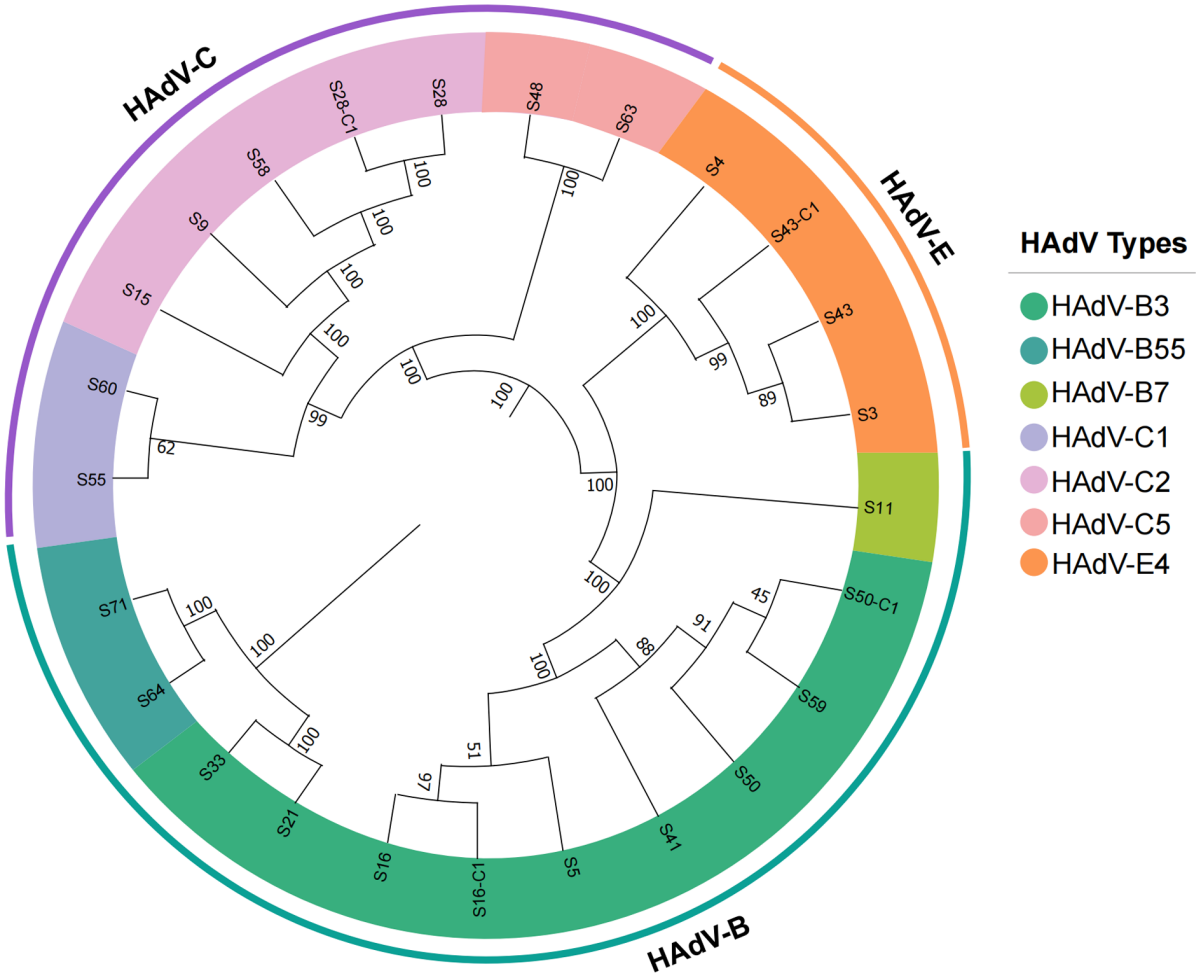
Phylogenetic analysis of all HAdV strains detected in this study based on complete genome data. The trees were constructed using the maximum likelihood method in MEGA11.0 with a bootstrap test of 1,000 replicates. According to specific HAdV species, each clade is highlighted using a color code.

Based on the similarity to the reference genomes, the 25 newly obtained strains were typed into 7 HAdV genotypes, most of which were species B and C. The whole-genome phylogenetic trees that were constructed for genotyping (Figure 2) enabled the classification of the seven genotypes as follows: HAdV-B3 (*n* = 9), B7 (*n* = 1), B55 (*n* = 2); HAdV-C1 (*n* = 2), C2 (*n* = 6), C5 (*n* = 1); and HAdV-E (*n* = 4). The main epidemic types were HAdV-B3 (36%, 9/25), HAdV-C2 (24%, 6/25), and HAdV-E4 (16%, 4/25). All sequences belonging to the same genotype clustered together with convincing bootstrap support. Despite the limited sample size, we detected multiple genotypes, which indicates the diversity of HAdVs in Hubei Province.

### 3.5. Reference-dependent phylogenetic and recombination analyses of HAdV-B species

**Figure 3:**
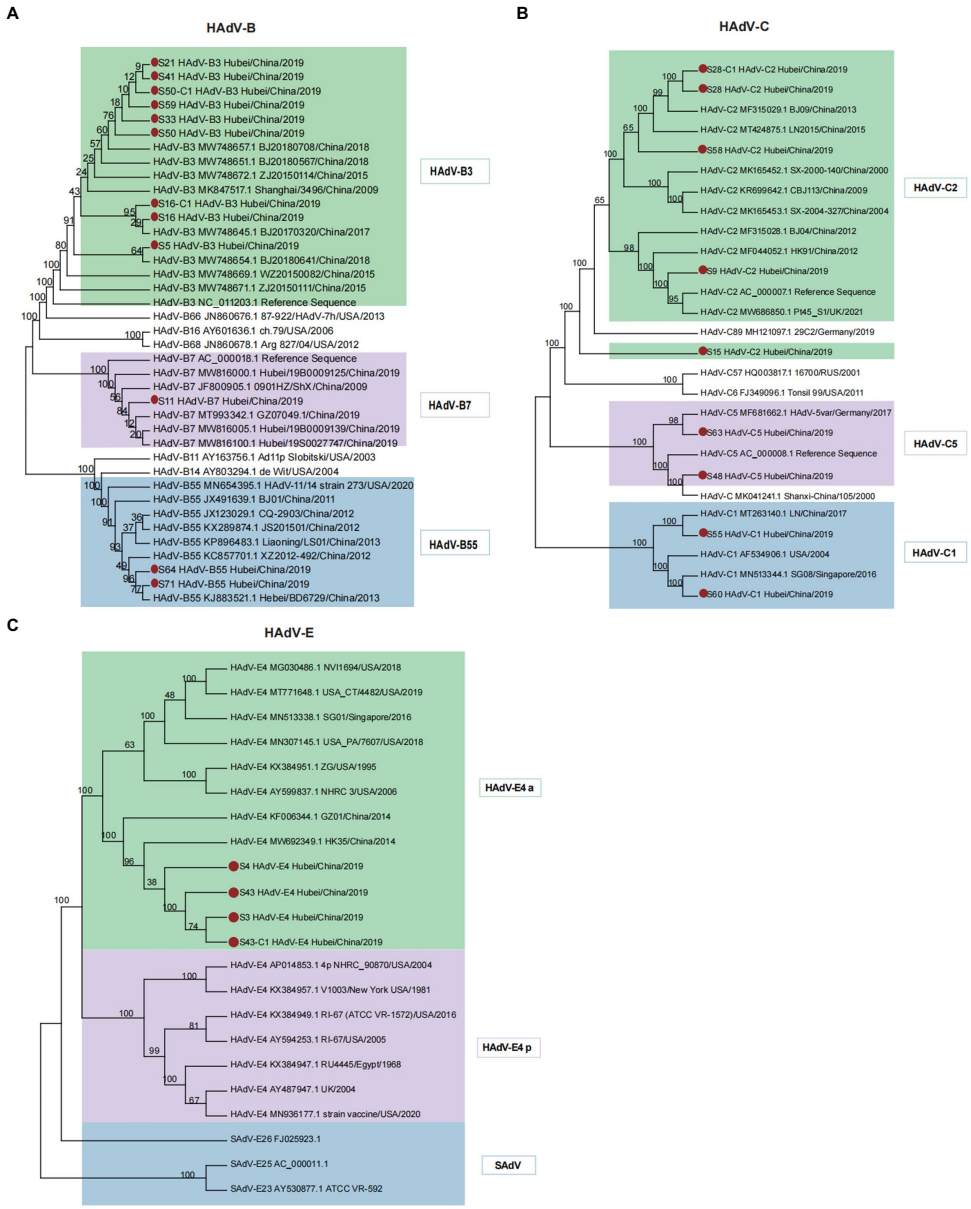
Phylogenetic analysis of HAdV-B, C, and E using the maximum likelihood method. **(A)** Phylogenetic tree of HAdV-B isolates. Different colored branches represent HAdVs isolated from different genotypes, designated HAdV-B3, B7, and B55. **(B)** Phylogenetic tree of HAdV-C isolates. Diverse colored branches represent HAdVs isolated from different genotypes, designated as HAdV-C1, C2, and C5. **(C)** Phylogenetic tree of HAdV-E isolates. Only one genotype (HAdV-E4) was identified. Two separable HAdV-E4 evolutionary lineages, prototype (p)-like and a-like, are highlighted in different colors. SAdVs were analyzed together to determine the phylogenetic relationships of HAdV-E4. Red dots denote new isolates collected in this study.

In this study, 12 cases were typed as B species. They diverged into separate clusters, including HAdV-B3, HAdV-B7, and HAdV-B55, together with the existing HAdV-B strains (Figure 3A). We observed that the newly identified HAdV-B3 strains (S21, S41, S50, S59, and S33) showed a close evolutionary relationship with strains identified in Beijing in 2017–2018, suggesting the possibility of a viral cross-regional spread. The HAdV-B7 strains detected in this study were closely related to the previously reported B7 strains (MW816005.1 and MW816100.1) from Hubei Province. The phylogenetic analyzes of the capsid *fiber*, *hexon*, and *penton* genes reconfirmed the clustering results (Supplementary Figure S2). HAdV-B55, a recombinant strain originating from parental strains HAdV-B11 and HAdV-B14, has gradually become the leading cause of community-acquired pneumonia in China (Wang et al., 2017). The S64 and S71 strains were grouped with the HAdV-B55 reference sequences, suggesting their potential as recombinant strains.

We also found that strain S64 originated from parental strains HAdV-B14 (AY803294.1) and HAdV-B11 (AF532578.1), as demonstrated through the recombination analysis (Supplementary Figure S3A). A high level of genome identity (98.77%) between strains S64 and HAdV-B14 was observed across the whole genome, except for the partial *hexon* gene (nucleotide gene location 18,661–19,611), which showed high similarity with HAdV-11 (Supplementary Figures S2, S3B). A similar recombination event was observed for strain S71 (Supplementary Figures S3C,D). Therefore, strains S64 and S71 identified in this study may have evolved from recombination between HAdV-B14 and HAdV-B11.

### 3.6. Reference-dependent phylogenetic and recombination analyses of HAdV-C species

The nine newly isolated strains were nested within the HAdV-C1, HAdV-C2, and HAdV-C5 genotypes with 100% bootstrap support (Figure 3B). Phylogenetic trees of the *hexon*, *penton*, and *fiber* genes were in agreement with the clustering pattern of the complete genomic sequences (Supplementary Figure S4). Strain S15, typed as C2 based on a similarity alignment to the reference genome, formed a separate cluster with other HAdV-C2 strains. Strain S28 was clustered with the Beijing HAdV-C2 strain (human/CHN/BJ09/MF315029/2013) with significant bootstrap support (99%), which was characterized by recombination of the HAdV-C1, HAdV-C5, and CBJ113 strains (Mao et al., 2017). Furthermore, recombination analyzes revealed that strain S28 (Supplementary Figure S3E) shared similar recombination patterns with BJ09, indicating that it originated from the parental strains HAdV-C1 (JX173083.1), HAdV-C5 (KF268199.1), and CBJ113 (KR699642.1). The pairwise whole-genome alignment indicated that strain S28 exhibited the most remarkable similarity to HAdV-CBJ113 across the entire genome, with 98.85% similarity in most genomic regions (Supplementary Figure S3F). Compared with HAdV-C1 and HAdV-C5, S28 displayed 97.20 and 96.42% similarity, respectively.

### 3.7. Reference-dependent phylogenetic analysis of HAdV-E species

In contrast to HAdV-C, the whole genomes of the new HAdV-E isolates (S3, S4, S43, and S43-C1) from Hubei were grouped within E4 with 100% bootstrap support (Figure 3C). HAdV-E4 has been previously classified as two separate evolutionary lineages: prototype (p)-like and a-like based on intratypic genetic variability (Li and Wadell, 1988). The a-like lineage (HAdV-E4 a) showed a relatively faster evolutionary rate than the p-like lineage because of a higher mutation frequency, indicating a broader host range and stronger transmissibility. In the present study, newly isolated HAdV-E4 strains clustered with a group of HAdV-E4 a-like strains from different countries but showed a closer evolutionary relationship with Chinese strains. The phylogenetic trees constructed for the *hexon*, *penton*, and *fiber* genes (Supplementary Figure S5) enabled the identification of different clusters. The phylogenetic clade of the SAdV genomes supported the previously formulated hypothesis of a zoonotic origin for HAdV-E4 (100% bootstrap support). SAdV-26 shared the closest relationship with HAdV-E4.

### 3.8. Nucleotide identity and diversity analysis

**Figure 4:**
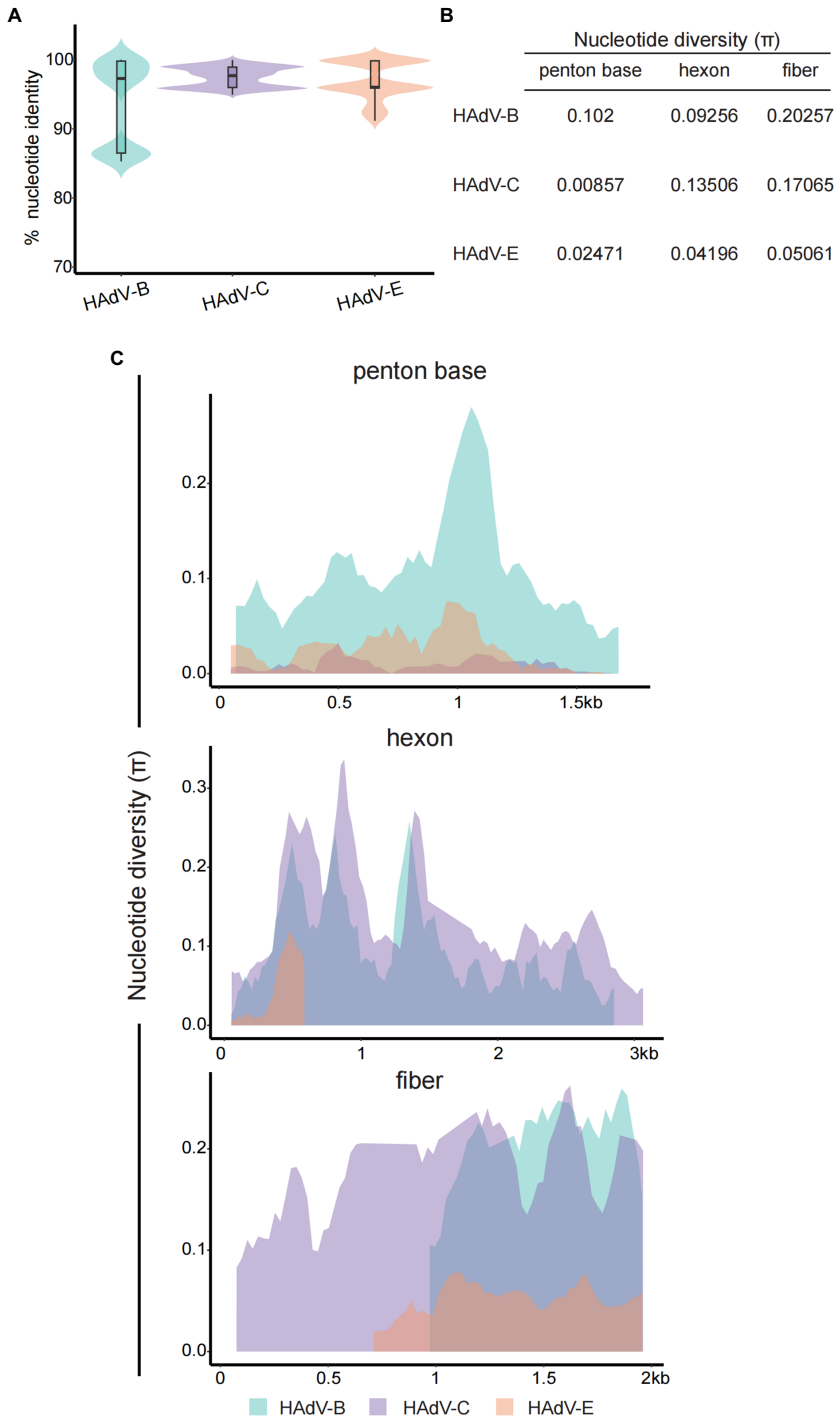
Nucleotide sequence identity and diversity for HAdV-B, C, and E. **(A)** Violin plot for percent nucleotide identity of different HAdV genotypes at the whole genome level. The box-plot in each violin displays the median, lower, and upper quartiles. The straight lines outside the box show the minimum and maximum values. **(B)** Average nucleotide diversity (π) statistics of the three major capsid genes between different HAdV genotypes. **(C)** Sliding window plot of the nucleotide diversity (π) per site (excluding gaps) showing the level of genetic diversity among the *penton*, *hexon*, and *fiber* genes of different HAdV genotypes. The π values were calculated on DnaSP v6 with 100 bp window length and 25 bp step size. HAdV-B and-E had shorter fiber genes than HAdV-C, and the aligning data was displayed right-aligned.

The percentage nucleotide identity of HAdVs’ whole genome sequences revealed a high level of genetic conservation within isolates from HAdV-B, C, or E genotypes. The average nucleotide identities of HAdV-B, C, and E were 93.52, 97.49, and 96.99%, respectively (Figure 4A). Three major capsid genes (*penton*, *hexon*, and *fiber*) were hotspot regions for homologous recombination and recognized as one major factor for HAdV genome diversity and viral evolution (Madisch et al., 2007; Xie et al., 2013). Average nucleotide diversity (π) statistics showed that the HAdV-C *penton* gene was relatively conserved (Figure 4B), reaffirming the previous results for HAdV-C species reported in the literature (Walsh et al., 2011). While the lowest diversity of *hexon* and *fiber* genes was observed in the HAdV-E genotype (Figure 4B), which may be explained by only one type (E4) classified within HAdV-E genotype. To further investigate the nucleotide differences per site along the capsid genes, we performed sliding window analysis among different HAdV genotypes (Figure 4C). For the HAdV-B genotype, divergence across the *penton* gene was generally higher, especially in two distinct hypervariable regions (HVR-1, location: about 450 ~ 510 bp; HVR-2, location: about 897 ~ 1,086 bp). In contrast, the *penton* gene of HAdV-C and E were relatively conserved, compatible with data in Figure 4B. The hexon protein contains two hypervariable loops (HVL1 and HVL2) domains that form the type-specific epitopes recognized by neutralizing antibodies (Bruder et al., 2012; Haque et al., 2018). In the present study, the nucleotide divergence of the *hexon* gene for HAdV-B and C was significantly higher than HAdV-E genotype with two larger peaks in 405 ~ 912 bp and 1,221 ~ 1,356 bp. Intriguingly, the diversity of HAdV-E *hexon* gene was close to zero from 600 bp. The fiber protein contains a critical C-terminal knob that constitutes the gamma epitope for hemagglutination inhibition and determines the cell tropism. The *fiber* gene showed higher variation than *hexon* and *penton* genes in all three HAdV genotypes. Notably, the gene length of *fiber* in HAdV-E and HAdV-B was shorter than in HAdV-C.

## 4. Discussion

Adenoviruses are the most frequently used vectors for delivering foreign genes or vaccine antigens and have been widely used for gene therapy, oncolytic virus therapy, and vaccine development, such as the Pfizer-BioNTech BNT162b2 vaccine against SARS-CoV-2 (Wold and Toth, 2013; Mendonça et al., 2021; Wang et al., 2021). Knowledge of whole adenoviral genomes is essential for constructing adenoviral vectors for downstream clinical applications. In this study, 25 adenovirus genome sequences were identified, which may be used as HAdV reference sequences to facilitate adenovirus related research. A total of 7 genotypes were identified from the limited 25 samples, indicating a high diversity of HAdVs in Hubei Province. For species B, newly identified HAdV-B3 isolates in Hubei have begun to form new distinct clusters, despite the similarity with strains from Beijing. This suggests the possibility of local spread, which could lead to widespread epidemiology. The newly emerged HAdV clade should be closely followed and monitored. With regard to strains S50 and S50-C1, their phylogenetic relationship remains controversial because of the different cluster groups (Figure 3A). Soltis et al. reported that low bootstrap values between nodes with a small number of characters can always be observed in closely related species that have not diverged extensively (Soltis and Soltis, 2003). Therefore, strains S21, S41, S50, S59, and S33 are most likely recently divergent strains with a very close evolutionary relationship.

Emerging pathogens pose a significant threat to global public health. Acute hepatitis of unknown etiology among children has recently attracted special attention worldwide. Although the causes of the disease are unclear, adenoviruses and SARS-CoV-2 have become the major focuses of investigations (Patel et al., 2022). In the European region, 53.1% of cases were reported positive for adenovirus and 64.7% positive rate in England, of which, the main suspect is adenovirus subtype 41 (UK Health Security Agency, 2022; World Health Organization, 2022). The monkeypox virus, a member of the *Orthopoxvirus* genus in the family *Poxviridae*, is also a double-stranded DNA virus that was first reported in Central Africa in 1970 (Breman et al., 1980). The 2022 outbreak of monkeypox involving multiple countries in both endemic and nonendemic regions has generated significant international concern (Titanji et al., 2022). As of October 25, 2022, 79,641 laboratory confirmed and 1,495 probable cases, including 51 deaths, have been reported to the World Health Organization.^2^ The rapid spread of monkeypox and acute hepatitis of unknown etiology highlights the importance of global epidemiological surveillance of microbial threats, including HAdV. In addition, HAdV surveillance has attracted increasing attention because of the continual emergence of new recombinant adenovirus strains, which are one of the main drivers of virus evolution.

The recombinant lineage 1 of HAdV-C has remained the domestic strain circulating in mainland China for decades, which includes CBJ113/China/2009 (KR699642.1), BJ09/China/2013 (MF315029.1), SX-2000-140/China/2000 (MK165452.1), and SX-2004-327/China/2004 (MK165453.1), all of which share the highest sequence similarity with the HAdV-C2 (NC_001405.1) prototype strain, especially in the major capsid genes (Yang et al., 2019). Consistent with the results of a previous report (Yang et al., 2019), the recombinant strains identified in our study (S28) were grouped within the HAdV-C2 cluster. The frequent recombination events between the HAdV-C types may be a significant driving force for the molecular evolution of HAdV-C. The closest reference strain, Shanxi-China/105/2000 (MK041241.1), mapping to S48, displays an unclear genotype in the NCBI database. Our analysis indicated that MK041241.1 is very close to the HAdV-C5 reference sequence (AC_000008.1) and forms a distinct cluster with the HAdV-C5 strains with strong bootstrap support (100%).

Nucleotide identity and diversity analysis is one of the most robust measurements of genomic relatedness and has been applied in studying the evolutionary relationships. By comparing and contrasting the genomes or capsid genes among different HAdV genotypes, we obtained a deeper insight into the phylogenetic relationships of the 25 isolates in this study. The high nucleotide identity within HAdV-B, C, and E genotypes agrees with the high conservation of the dsDNA virus, but is interrupted by hypervariability at the three major capsid genes, consistent with the consensus that homologous recombination frequently occurs in these hotspot gene regions. Homologous recombination plays a primary role in generating genome diversity and contributing to viral elevation. Our study noted marked differences in *penton*, *hexon*, and *fiber* genes across genotypes. Pronounced hypervariable regions in *penton* and *hexon* genes were observed in the nucleotide diversity sliding plots, accordant with the previous study. The *hexon* gene of HAdV-E presents a solitary peak without continuous π values from ~600 bp.We speculated that the single E4 type within HAdV-E genotype and fewer reference sequences caused this interruption. Certainly, substantial HAdV-E sequences are required to justify this hypothesis in the future.

mNGS analysis facilitates precise pathogen detection and identification for the clinical diagnosis of infections. mNGS can be used to simultaneously detect multiple pathogens, which is of great importance for diagnosing unknown infections, especially in cases with negative results obtained through conventional methods of pathogen detection. Huang et al. reported that mNGS detected disease-associated microbes in 94.49% of patients with pulmonary infection who had negative results obtained *via* conventional methods (Huang J. et al., 2020). Similarly, Chen et al. reported that the detection rate of pathogenic bacteria *via* mNGS was significantly higher than that by the culture method (65.0% vs. 20.0%) in patients with lower respiratory tract infection (Chen H. et al., 2020). Therefore, mNGS may become a routine diagnostic test for clinical infection, partially replacing the conventional pathogen culture method. In the present study, a wide variety of pathogens other than HAdVs were detected in the clinical mNGS samples compared with cell culture samples, including *Prevotella*, *Veillonella*, and *Streptococcus*. However, the interpretation of mNGS data requires further study, especially the detection of pathogenic bacteria, colonizing bacteria, and mixtures of normal oral microbiota.

To the best of our knowledge, this is the first mNGS study to systematically describe clinical HAdV genotypes and perform phylogenetic characterization using the GenoLab M platform. In our previous study, GenoLab M showed comparable performance metrics to WGS and whole-exome sequencing (Li et al., 2022), transcriptomics, and LncRNA (Liu et al., 2021) applications when compared in parallel with the Illumina NovaSeq sequencing platform. In the present retrospective study, we compared the applicability of the GenoLab M platform with that of the NextSeq 550 platform in mNGS. Our findings demonstrated interplatform consistency in data quality, assembly results, and microbial annotation, indicating the potential of GenoLab M as an alternative to NextSeq 550 in mNGS for HAdV characterization and surveillance. Although the mapping rate of the viral genome assembled from short-read-length mNGS data can achieve 90% alignment with the reference sequence, there remain certain unmatched gaps. In the future, we plan to combine the nanopore sequencing platform (Oxford Nanopore Technologies) with GenoLab M for the genomic characterization and phylogenetic analyzes of HAdVs. Nanopore sequencing enables the generation of long-read viral genomes from pure cultures and metagenomic samples. Meanwhile, high-throughput GenoLab M sequencing could obtain accurate mutation information and correct errors, resulting in an increased understanding of HAdV genotypes and recombination.

In conclusion, our findings indicate that culture-independent mNGS is a reliable method with high sequencing quality and assembly accuracy and can be used for HAdV genotyping and genome research. The GenoLab M sequencer can be successfully used for mNGS. More in-depth genomic analyzes of HAdV strains circulating worldwide are needed to increase our understanding of the genetic diversity of HAdVs. In addition, the sequencing dataset established in this study can be used as a reference for future studies employing complete genomic sequencing of HAdV strains.

## Data availability statement

The datasets presented in this study can be found in online repositories. The names of the repository/repositories and accession number(s) can be found at: https://db.cngb.org/, CNP0003602. Because it involves the protection of human genetic resources, the datasets from patients are not publicly available but are available from the corresponding author on reasonable request and upon completion of any necessary interinstitutional Materials Transfer Agreement.

## Ethics statement

The studies involving human participants were reviewed and approved by Ethics board of the Hubei Provincial Center for Disease Control and Prevention. Written informed consent from the participants' legal guardian/next of kin was not required to participate in this study in accordance with the national legislation and the institutional requirements.

## Author contributions

BF, JL, and YL: conceptualization, investigation, and writing and editing. TY, XY, and XL: sample collection and DNA extract and library construction. LD and QY: sequencing. XZ and WY: bioinformatics analysis. LS and LL: directing the project. All authors contributed to the article and approved the submitted version.

## Funding

This study was supported by the project of Key R&D Program of Hubei, Science and Technology Department of Hubei Province (no. 2020BCA090).

## Conflict of interest

Authors JL, YL, LD, XZ, WY, QY, and LS were employed by GeneMind Biosciences Company Limited, Shenzhen, China.

The remaining authors declare that the research was conducted in the absence of any commercial or financial relationships that could be construed as a potential conflict of interest.

## Publisher’s note

All claims expressed in this article are solely those of the authors and do not necessarily represent those of their affiliated organizations, or those of the publisher, the editors and the reviewers. Any product that may be evaluated in this article, or claim that may be made by its manufacturer, is not guaranteed or endorsed by the publisher.

## Footnotes

^1^https://genome-idx.s3.amazonaws.com/kraken/k2_standard_20210517.tar.gz

^2^https://worldhealthorg.shinyapps.io/mpx_global/
