# Supplementary material for: Genetic characterization of human adenoviruses in patients using metagenomic next-generation sequencing in Hubei, China, from 2018 to 2019
Source: Front Microbiol. 2023 Mar 16;14:1153728. doi: 10.3389/fmicb.2023.1153728 (PMC10060807; doi:10.3389/fmicb.2023.1153728)
Supplement: Supplementary file 1 [file Table_1.DOC]

Table S1. Patient characteristics in this study.

| **Sample** | **Age-years** | **Sex** | **Time** | **Sample type** | **Ct value** |
| --- | --- | --- | --- | --- | --- |
| S3 | 6 | Male | 20190121 | Nasopharyngeal swab | 23.66 |
| S4 | 7 | Male | 20190128 | Nasopharyngeal swab | 20.33 |
| S9 | 1 | Male | 20190320 | Nasopharyngeal swab | 15.56 |
| S16 | 2 | Male | 20190507 | Nasopharyngeal swab | 16.34 |
| S41 | 11 | Male | 20190813 | Nasopharyngeal swab | 17.38 |
| S5 | 1 | Male | 20190227 | Nasopharyngeal swab | 17.68 |
| S15 | 3 | Male | 20190507 | Nasopharyngeal swab | 18.26 |
| S33 | 3 | Male | 20190626 | Nasopharyngeal swab | 18.85 |
| S21 | 5 | Male | 20190512 | Nasopharyngeal swab | 19.27 |
| S28 | 1 | Female | 20190528 | Nasopharyngeal swab | 19.6 |
| S59 | 9 | Male | 20191119 | Nasopharyngeal swab | 19.85 |
| S60 | 7 | Female | 20191124 | Nasopharyngeal swab | 20.49 |
| S48 | NC | NC | NC | Nasopharyngeal swab | 20.87 |
| S55 | 2 | Female | 20181126 | Nasopharyngeal swab | 20.9 |
| S58 | 1 | Male | 20191119 | Nasopharyngeal swab | 21.09 |
| S63 | 3 | Male | 20191210 | Nasopharyngeal swab | 21.56 |
| S11 | 4 | Female | 20190324 | Nasopharyngeal swab | 21.58 |
| S64 | NC | NC | NC | Nasopharyngeal swab | 21.82 |
| S43 | 6 | Female | 20190917 | Nasopharyngeal swab | 23.5 |
| S71 | NC | NC | NC | Nasopharyngeal swab | 26.36 |
| S50 | 2 | Male | 20181116 | Nasopharyngeal swab | 32.13 |
| S16-C1 | 2 | Male | 20190507 | Culture virus | 19.66 |
| S28-C1 | 1 | Female | 20190528 | Culture virus | 21.78 |
| S43-C1 | 6 | Female | 20190917 | Culture virus | 24.76 |
| S50-C1 | 2 | Male | 20181116 | Culture virus | 26.36 |

Note：

NC, Not collected

Ct, cycle threshold
