# Supplementary material for: Genetic characterization of human adenoviruses in patients using metagenomic next-generation sequencing in Hubei, China, from 2018 to 2019
Source: Front Microbiol. 2023 Mar 16;14:1153728. doi: 10.3389/fmicb.2023.1153728 (PMC10060807; doi:10.3389/fmicb.2023.1153728)
Supplement: Supplementary file 2 [file Table_2.DOC]

Table S2. General sequencing characteristics of two platforms.

| **Sample** | **Clean bases (Gb)** | | **Q20 (%)** | | **Q30 (%)** | | **GC (%)** | | **Human ratio (%)** | | **Duplicated reads ratio (%)** | |
| --- | --- | --- | --- | --- | --- | --- | --- | --- | --- | --- | --- | --- |
|  | **GL** | **NS** | **GL** | **NS** | **GL** | **NS** | **GL** | **NS** | **GL** | **NS** | **GL** | **NS** |
| S11 | 1.78 | 1.76 | 97.11 | 90.80 | 92.72 | 85.39 | 44.08 | 45.23 | 51.49 | 51.15 | 0.87 | 0.98 |
| S15 | 1.77 | 1.76 | 97.45 | 88.52 | 93.67 | 81.86 | 41.31 | 41.94 | 38.81 | 37.78 | 0.48 | 0.14 |
| S16-C1 | 1.78 | 1.76 | 97.82 | 88.00 | 94.29 | 80.98 | 41.71 | 42.03 | 72.27 | 70.56 | 3.35 | 1.38 |
| S16 | 1.78 | 1.65 | 97.64 | 90.43 | 93.95 | 84.58 | 41.77 | 42.54 | 59.83 | 58.93 | 0.70 | 0.72 |
| S21 | 1.79 | 1.77 | 97.53 | 91.40 | 93.65 | 85.95 | 41.22 | 42.24 | 47.54 | 47.05 | 1.62 | 1.39 |
| S28-C1 | 1.79 | 1.54 | 98.00 | 90.75 | 94.68 | 85.01 | 42.02 | 42.99 | 81.86 | 80.15 | 2.92 | 2.69 |
| S28 | 1.78 | 1.76 | 96.24 | 88.29 | 90.73 | 81.51 | 41.26 | 42.03 | 58.14 | 58.25 | 0.31 | 0.16 |
| S3 | 1.79 | 1.76 | 95.94 | 89.54 | 90.00 | 83.29 | 41.56 | 42.59 | 67.07 | 67.82 | 0.56 | 0.52 |
| S33 | 1.79 | 1.59 | 97.77 | 90.19 | 93.97 | 84.01 | 41.05 | 41.98 | 2.74 | 2.58 | 2.97 | 1.45 |
| S4 | 1.78 | 1.70 | 97.51 | 90.41 | 93.50 | 84.91 | 45.49 | 47.88 | 23.24 | 20.87 | 14.23 | 10.65 |
| S41 | 1.78 | 1.76 | 96.86 | 89.03 | 92.23 | 82.77 | 40.83 | 41.75 | 68.03 | 67.04 | 0.50 | 0.42 |
| S43-C1 | 1.79 | 1.76 | 96.56 | 88.85 | 91.33 | 82.17 | 40.20 | 40.87 | 76.49 | 77.24 | 0.38 | 0.27 |
| S43 | 1.79 | 1.78 | 97.18 | 91.53 | 92.58 | 86.15 | 42.90 | 44.98 | 6.70 | 6.16 | 2.77 | 2.19 |
| S48 | 1.79 | 1.68 | 97.11 | 90.02 | 92.66 | 83.92 | 40.92 | 42.06 | 72.31 | 71.56 | 0.41 | 0.68 |
| S5 | 1.79 | 1.76 | 96.18 | 88.55 | 90.50 | 81.75 | 39.49 | 40.27 | 61.55 | 61.51 | 0.28 | 0.24 |
| S50-C1 | 1.79 | 1.76 | 97.85 | 88.67 | 94.38 | 81.93 | 40.38 | 40.82 | 80.85 | 79.31 | 0.86 | 0.31 |
| S50 | 8.93 | 8.91 | 97.45 | 91.60 | 93.50 | 86.30 | 42.51 | 43.09 | 38.60 | 38.09 | 8.33 | 5.68 |
| S55 | 1.78 | 1.77 | 96.87 | 90.57 | 92.21 | 84.85 | 41.88 | 43.01 | 48.17 | 46.93 | 0.34 | 0.68 |
| S58 | 1.77 | 1.76 | 97.29 | 91.29 | 93.31 | 86.02 | 42.38 | 43.34 | 46.40 | 45.29 | 0.70 | 0.97 |
| S59 | 1.78 | 1.76 | 96.94 | 89.01 | 92.44 | 82.42 | 40.69 | 41.34 | 74.55 | 73.38 | 0.36 | 0.19 |
| S60 | 1.78 | 1.66 | 98.02 | 90.81 | 94.87 | 85.52 | 39.94 | 43.17 | 70.41 | 55.43 | 23.69 | 6.49 |
| S63 | 1.73 | 1.77 | 96.53 | 90.94 | 91.55 | 85.47 | 42.60 | 44.41 | 29.31 | 27.06 | 0.65 | 1.08 |
| S64 | 1.78 | 1.76 | 98.00 | 89.57 | 94.74 | 83.25 | 40.92 | 41.35 | 76.06 | 75.32 | 0.71 | 0.47 |
| S71 | 5.38 | 5.30 | 96.22 | 90.06 | 90.50 | 84.07 | 40.50 | 41.54 | 82.68 | 83.67 | 0.91 | 0.83 |
| S9 | 1.78 | 1.76 | 96.82 | 88.87 | 92.14 | 82.34 | 40.81 | 41.59 | 59.73 | 58.65 | 0.39 | 0.29 |

Note：

GL, GenoLab M

NS, NextSeq 550
