# Supplementary material for: Genetic characterization of human adenoviruses in patients using metagenomic next-generation sequencing in Hubei, China, from 2018 to 2019
Source: Front Microbiol. 2023 Mar 16;14:1153728. doi: 10.3389/fmicb.2023.1153728 (PMC10060807; doi:10.3389/fmicb.2023.1153728)
Supplement: Supplementary file 3 [file Table_3.DOC]

Table S3. General assembly statistics.

|  | **Reference** | **Reference GC (%)** | **GC (%)** | | **Mapping ratio (%)** | | **Average depth** | | **Coverage (%)** | |
| --- | --- | --- | --- | --- | --- | --- | --- | --- | --- | --- |
|
| **Sample** |  |  | **GL** | **NS** | **GL** | **NS** | **GL** | **NS** | **GL** | **NS** |
| S11 | MW816005.1 | 51.08 | 51.11 | 51.1 | 45.48 | 0.28 | 3,072 | 62 | 100 | 100 |
| S15 | MT424875.1 | 55.22 | 55.19 | 55.17 | 46.67 | 0.58 | 4,747 | 133 | 100 | 100 |
| S16-C1 | MW748645.1 | 50.89 | 50.9 | 50.87 | 78.02 | 25.9 | 5,812 | 3,181 | 100 | 100 |
| S16 | MW748645.1 | 50.89 | 50.88 | 50.88 | 1.05 | 1.05 | 178 | 178 | 100 | 100 |
| S21 | MW748657.1 | 50.89 | 50.92 | 50.88 | 29.71 | 33.39 | 2,092 | 2,965 | 100 | 100 |
| S28-C1 | MF315029.1 | 55.22 | 55.22 | 55.26 | 26.99 | 27.37 | 2,262 | 2,274 | 100 | 100 |
| S28 | MF315029.1 | 55.22 | 55.2 | 55.24 | 53.94 | 53.79 | 3,679 | 4,036 | 100 | 100 |
| S3 | KF006344.1 | 56.3 | 56.31 | 56.28 | 0.1 | 0.1 | 14 | 14 | 100 | 100 |
| S33 | MW748657.1 | 50.89 | 50.9 | 50.88 | 12.11 | 0.11 | 1,787 | 50 | 100 | 100 |
| S4 | KF006344.1 | 56.3 | 56.36 | 56.36 | 18.71 | 18.71 | 6,642 | 6,642 | 100 | 100 |
| S41 | MW748657.1 | 50.89 | 50.88 | 50.91 | 0.51 | 0.51 | 70 | 70 | 100 | 100 |
| S43-C1 | KF006344.1 | 56.3 | 54.89 | 56.25 | 71.84 | 68.03 | 3,539 | 2,755 | 100 | 100 |
| S43 | KF006344.1 | 56.3 | 56.33 | 56.33 | 0.07 | 0.07 | 31 | 31 | 100 | 100 |
| S48 | MK041241.1 | 55.17 | 55.15 | 55.21 | 0.72 | 0.72 | 83 | 83 | 100 | 100 |
| S5 | MW748654.1 | 50.88 | 50.85 | 50.88 | 0.3 | 0.3 | 49 | 49 | 100 | 100 |
| S50-C1 | MW748657.1 | 50.89 | 50.91 | 50.87 | 72.22 | 63.28 | 1,925 | 1,334 | 100 | 100 |
| S50 | MW748657.1 | 50.89 | 50.89 | 50.89 | 0.26 | 0.26 | 366 | 365 | 100 | 100 |
| S55 | MT263140.1 | 55.23 | 55.16 | 55.28 | 41.12 | 41.53 | 3,420 | 3,450 | 100 | 100 |
| S58 | MF315029.1 | 55.22 | 55.31 | 55.05 | 55.29 | 54.94 | 5,022 | 5,089 | 100 | 99 |
| S59 | MW748672.1 | 50.89 | 50.89 | 50.88 | 0.53 | 0.53 | 58 | 58 | 100 | 100 |
| S60 | MN513344.1 | 55.44 | 56.18 | 55.67 | 27.36 | 31.46 | 1,498 | 1,270 | 100 | 99 |
| S63 | MF681662.1 | 55.22 | 54.97 | 55.2 | 2.82 | 2.82 | 795 | 804 | 100 | 100 |
| S64 | KX289874.1 | 48.77 | 48.8 | 48.82 | 0.29 | 62.21 | 30 | 1,952 | 100 | 100 |
| S71 | KX289874.1 | 48.77 | 48.8 | 48.8 | 0.55 | 64.94 | 133 | 5,937 | 100 | 100 |
| S9 | MZ151863.1 | 55.19 | 55.15 | 55.22 | 51.05 | 0.61 | 3,123 | 98 | 100 | 100 |

Note：

GL, GenoLab M

NS, NextSeq 550
