# Supplementary figures and images for: Genetic characterization of human adenoviruses in patients using metagenomic next-generation sequencing in Hubei, China, from 2018 to 2019
Source: Front Microbiol. 2023 Mar 16;14:1153728. doi: 10.3389/fmicb.2023.1153728 (PMC10060807; doi:10.3389/fmicb.2023.1153728)

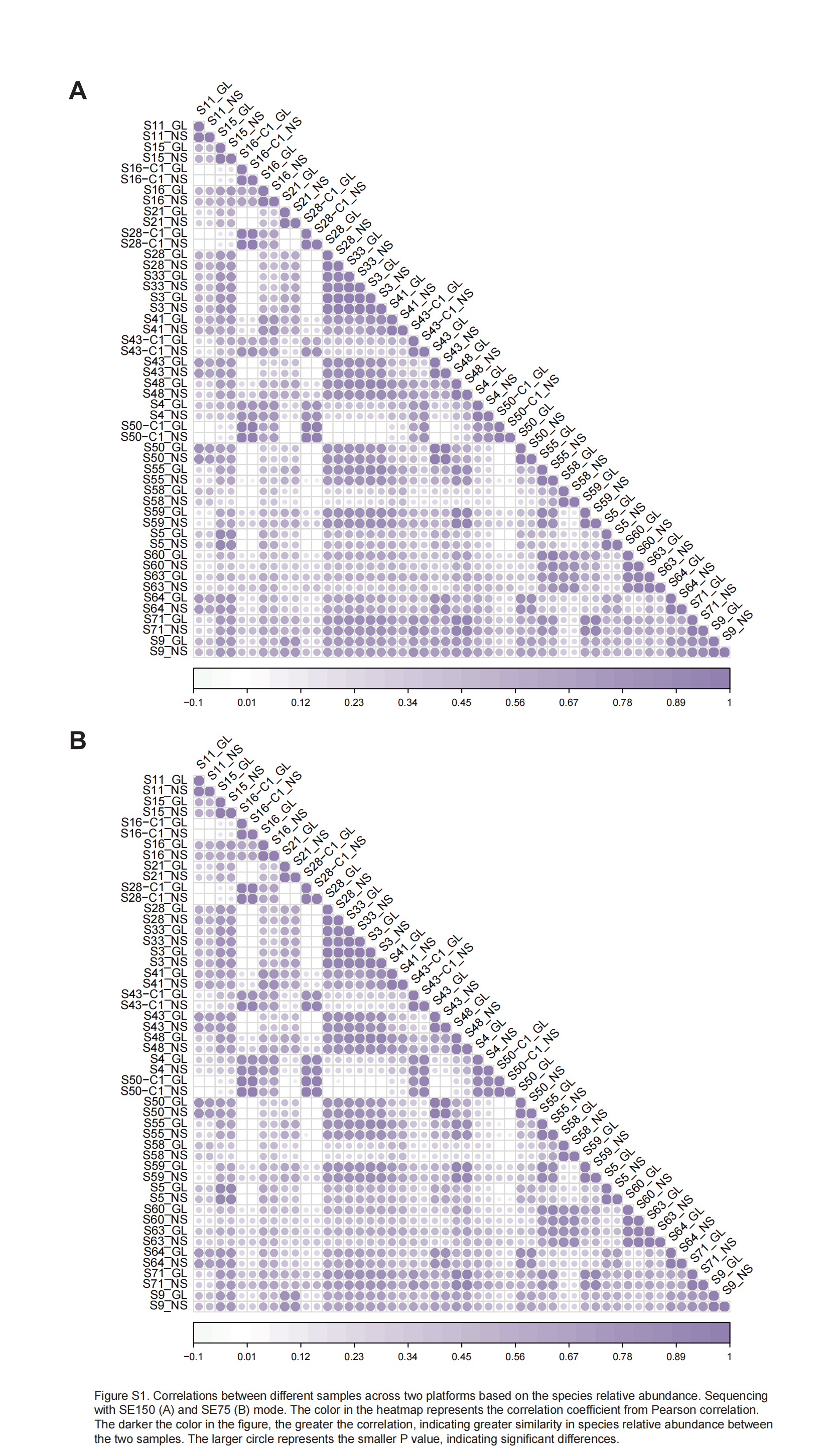

Supplement: Supplementary file 4 [file Image_1.TIF]

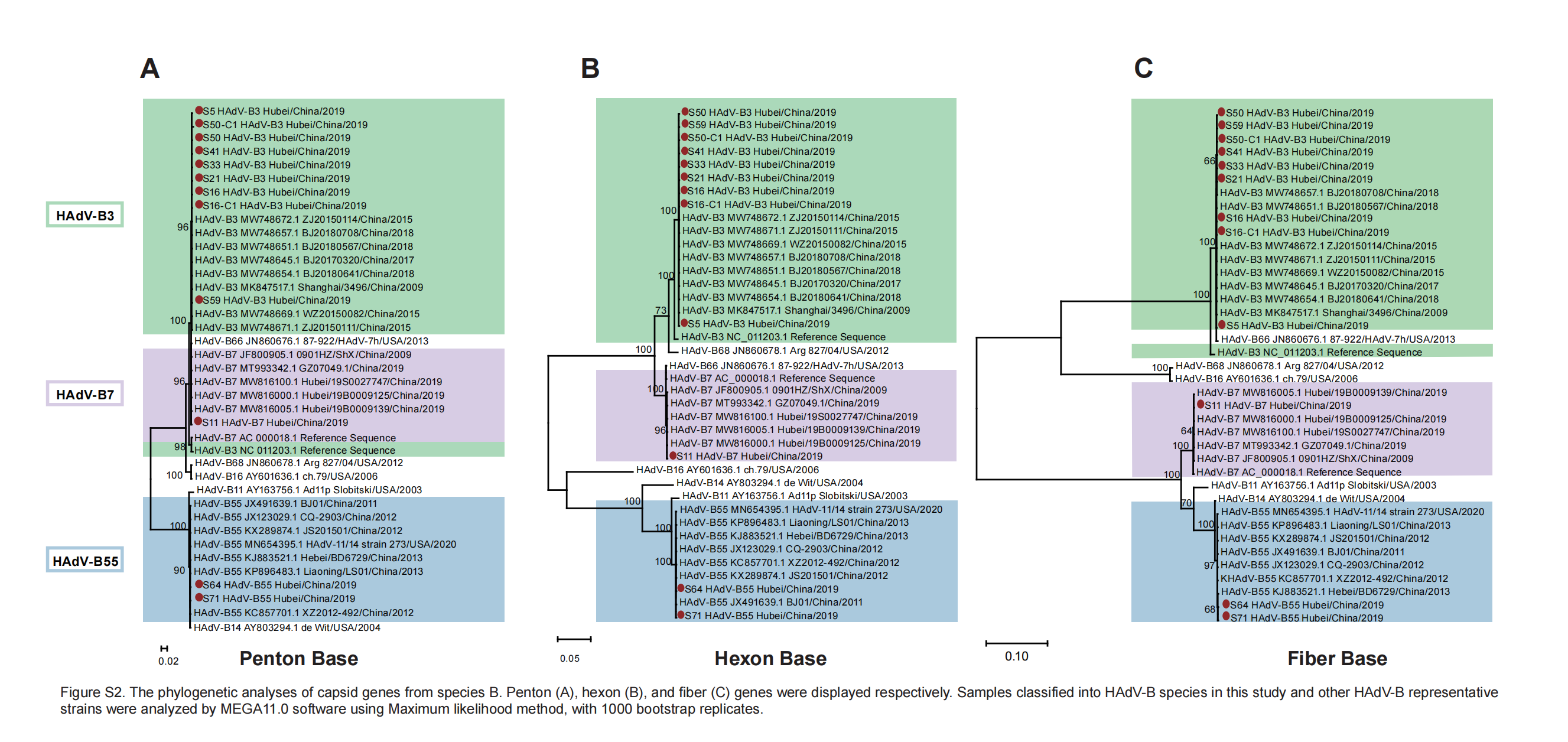

Supplement: Supplementary file 5 [file Image_2.TIF]

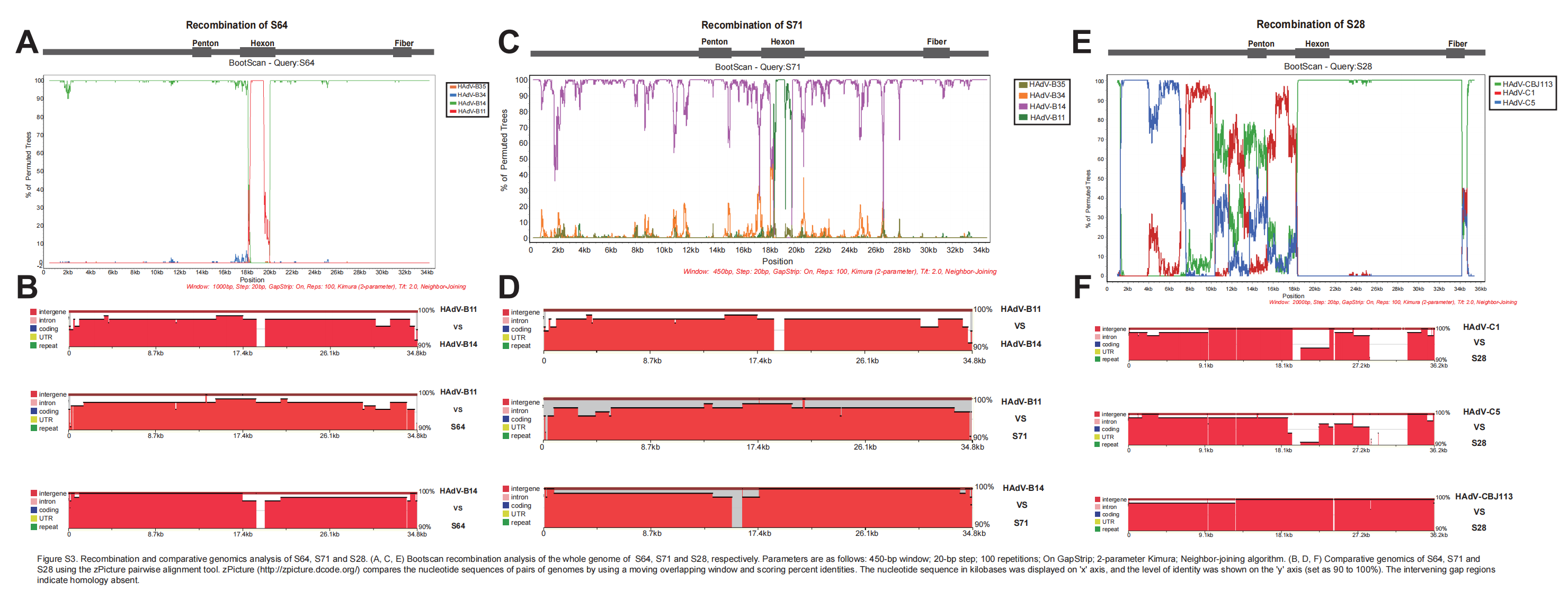

Supplement: Supplementary file 6 [file Image_3.TIF]

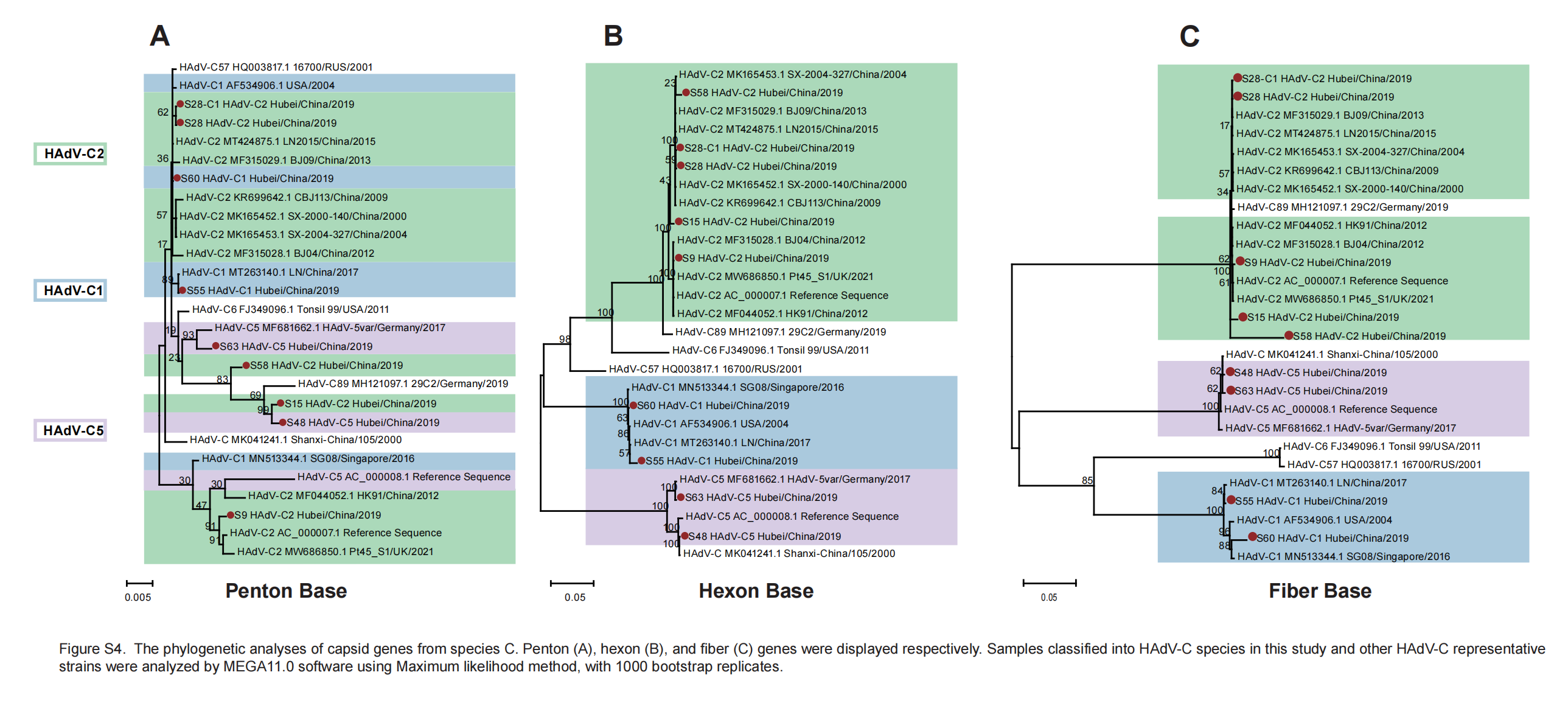

Supplement: Supplementary file 7 [file Image_4.TIF]

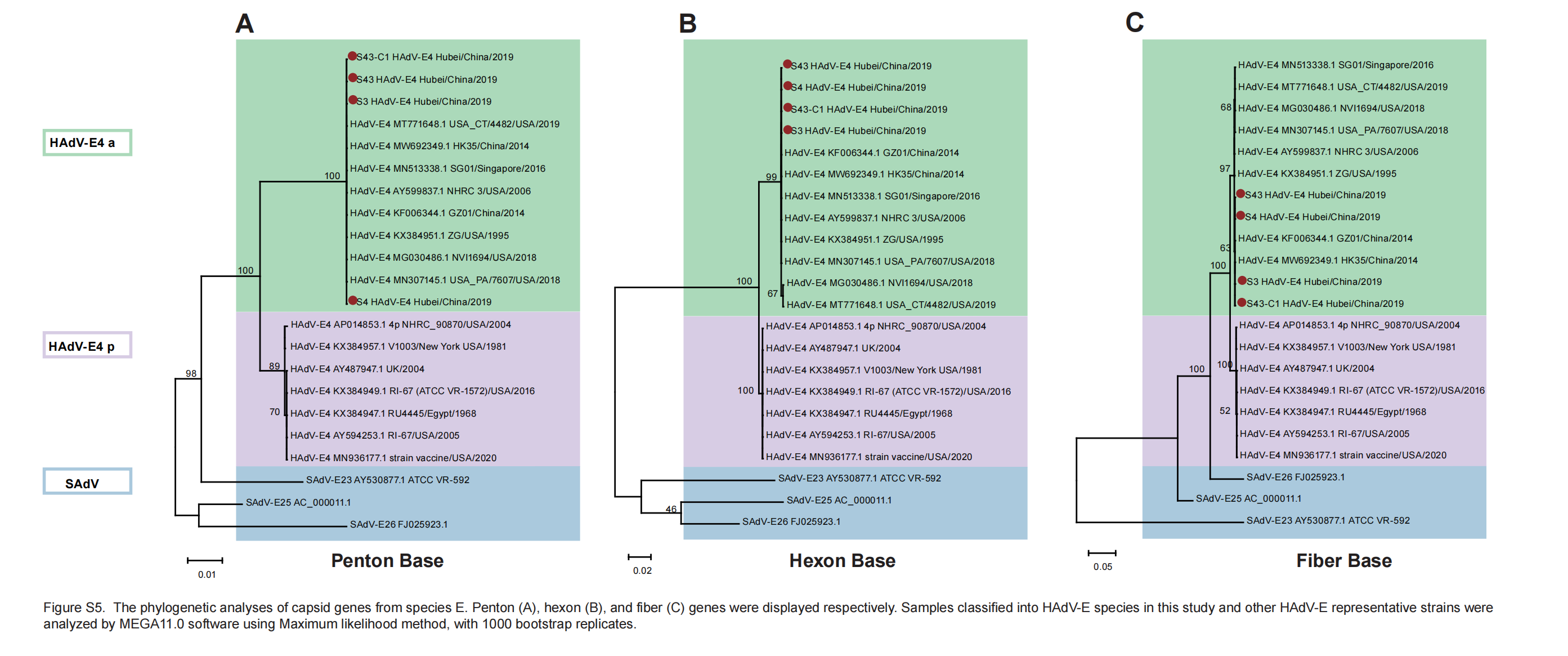

Supplement: Supplementary file 8 [file Image_5.TIF]
